# The genus *Apodrosus* Marshall, 1922 in Cuba (Coleoptera, Curculionidae, Entiminae, Polydrusini)

**DOI:** 10.3897/zookeys.679.12805

**Published:** 2017-06-12

**Authors:** Robert S. Anderson, Guanyang Zhang

**Affiliations:** 1 Research and Collection Division, Canadian Museum of Nature, PO Box 3443, Station D, Ottawa, ON. K1P 6P4, Canada; 2 School of Life Sciences, PO Box 874501, Arizona State University, Tempe, AZ. 85287, U.S.A.

**Keywords:** New species, weevils, West Indies, Caribbean, biodiversity

## Abstract

The genus *Apodrosus* Marshall is newly recorded for, and revised for Cuba. Nine new species are recognized as follows: *Apodrosus alberti* (type locality, Granma, Parque Nacional Pico Turquino), *A. alternatus* (type locality, Guantánamo, El Yunque), *A. franklyni* (type locality, Cienfuegos, Parque Nacional Pico San Juan), *A. griseus* (type locality, Santiago de Cuba, Siboney-Jutici Ecological Reserve), *A. mensurensis* (type locality, Holguin, Parque Nacional La Mensura-Piloto), *A. pseudoalternatus* (type locality, Matanzas, Varahicacos), *A. beckeli* (type locality, Guantánamo, 8 km W. Imias), *A. sandersoni* (type locality, Guantánamo, Loma Lafarola), and *A. zayasi* (type locality, Cienfuegos, Parque Nacional Pico San Juan). A key for their identification, descriptions, summaries of natural history information and data on distributions are presented. A molecular phylogeny based on 11 species of *Apodrosus* from Cuba, Hispaniola and Puerto Rico is reconstructed. A sister group relationship between *Polydrusus* and *Apodrosus* is recovered with a limited sampling of the former genus. The monophyly of *Apodrosus* is recovered with strong support. Cuban *Apodrosus* are not monophyletic. Five of the six sampled Cuban species form a clade, sister to an undescribed *Apodrosus* species from the Dominican Republic; and, *Apodrosus alternatus* is sister to *A. quisqueyanus* Girón & Franz, 2010, a species from the Dominican Republic. Biogeographic implications for Cuban species are discussed in light of the phylogeny.

## Introduction

The genus *Apodrosus* Marshall, 1922 was recently comprehensively revised by Girón and Franz (2010). They documented the presence of 13 species throughout the Caribbean, eleven of which they described as new. No species of the genus were known from Cuba. Following three recent (2012, 2013, 2014) expeditions to Cuba we have now recorded nine additional endemic species of the genus from throughout the country. These species are described herein.

Girón and Franz (2010) also presented a morphological phylogeny and biogeographic analysis of the genus with two main clades represented; the Hispaniolan/Puerto Rican *A. artus*-*A. wolcotti* clade from elevations over 1000 m and the more speciose and widespread western Caribbean *A. eximius*-*A. empherefasciatus* clade from arid, lower-elevation, coastal habitats. Here, we reinterpret their results based on a limited molecular phylogeny incorporating data from 11 species of the genus from Cuba, Hispaniola and Puerto Rico. Host plant associations continue to remain uncertain.

The phylogenetic study by Girón and Franz (2010) questioned the placement of *Apodrosus* in Polydrusini. At present, we do not address this issue as any changes in tribal placement would require definition of the pertinent tribes of Entiminae and this is well beyond the scope of this study.

## Materials and methods

The approach used in this study follows that of Girón and Franz (2010) for ease of comparison of species from Cuba with the rest of the genus. A differential diagnosis is presented allowing for separation from other Cuban species only. Cuban species are considered distinct based on comparison of male genitalia with figures in Girón and Franz (2010) and on geography. Specimens are deposited in the Hasbrouck Insect Collection of Arizona State University, Tempe, AZ (ASUHIC); Canadian Museum of Nature, Ottawa, Canada (CMNC); Charles W. O’Brien Collection, Green Valley, AZ (CWOB); Illinois Natural History Survey, Champaign, IL (INHS); and United States National Museum, Washington D.C. (USNM). Species descriptions were prepared by RSA and phylogenetic analysis by GYZ. Species are listed in alphabetical order. Latitude and longitude data are given in decimal degrees. A map of Cuban *Apodrosus* is included.

Phylogenies of *Apodrosus* species and their allies were reconstructed using molecular data. Two successive analyses were conducted, first with a broad outgroup sample, and the second focusing on just *Apodrosus* species (details of samples including geography, vouchering and Genbank numbers are provided in Suppl. material 1). Eleven species of *Apodrosus* were sampled for molecular data, with six from Cuba and five from Puerto Rico or Hispaniola (represented by the Dominican Republic). Other species of *Apodrosus* were not sampled due to a lack of DNA-quality specimens or failed molecular experiments. In the first analysis, sequences of eight species of *Polydrusus* and one species of *Pachyrhinus*, all Palearctic, were downloaded from Genbank. These were included to test the relationship between *Apodrosus* and *Polydrusus*, both currently placed in Polydrusini (Alonso-Zarazaga & Lyal, 1999). Representatives of New World *Polydrusus* were not sampled due to absence of published data or fresh specimens. Two genes (Cytochrome c Oxidase subunit I [COI], and Cytochrome c Oxidase subunit II [COII]), are available for *Polydrusus* and *Pachyrhinus*. Eleven other species of various other Neotropical entimines were also included to position Polydrusini as sampled in the current study in a broader phylogenetic context, an exercise hitherto not performed. This selection represents a broad coverage of Caribbean entimine diversity at the tribal level, but is also limited by sample availability. Girón and Franz (2010) placed *Anypotactus bicaudatus* as the sister of *Apodrosus* sampled in that study. Due to lack of DNA-quality material, this genus is not sampled in the current study. *Anthonomus grandis* was used to root the 33-taxon phylogeny. This phylogeny recovered a sister relationship between Polydrusus (Pachyrhinus) and *Apodrosus* (Suppl. material 2). In a second analysis, only species of *Apodrosus* and a *Polydrusus* (as root) were retained and this phylogeny is presented in the main text of the article. DNA extractions were performed using the Qiagen DNeasy Blood and Tissue Kit (Qiagen). The right hind leg was excised from a specimen and extracted. Six mitochondrial, nuclear, protein-coding or ribosomal gene fragments – COI, COII, 12s, Arginine Kinase (AK), Elongation Factor 1-alpha (Ef 1-α), and 28s (D2-D4 regions) – were amplified with polymerase chain reaction using EmeraldAmp MAX PCR Master Mix in an Eppendorf vapo protect thermal cycler. Primer and PCR programming information is the same as in Zhang et al. (2017). DNA sequencing was performed at the Arizona State University DNA Laboratory on an Applied Biosystems 3730 capillary sequencer. DNA sequences were edited with the software Geneious R7 and uploaded to Genbank. The software program MAFFT (Katoh and Standley 2013) was used to align DNA sequences, applying the auto alignment strategy for protein coding genes (COI, COII, AK and Ef-1α) and L-INS-i for ribosomal genes (12s and 28s). The aligned, individual gene data sets were concatenated using SequenceMatrix (Vaidya et al. 2011). A matrix of 33 terminals and 4539 aligned nucleotide sites was generated for the first analysis and another with 13 terminals and 4494 sites for the second analysis. The maximum likelihood method was used to reconstruct phylogenies using the program RAxML (Stamatakis 2014) via the CIPRES (www.phylo.org) supercomputer cluster (Miller et al. 2010). The analysis was partitioned with each gene fragment considered a different partition.

## Taxonomy

### Apodrosus

Taxon classificationAnimaliaColeopteraCurculionidae

Marshall, 1922

Apodrosus Marshall, 1922: 59.
Apodrusus Marshall (in Wolcott 1924: 130, error).

#### Gender masculine.

Type species *Apodrosus wolcotti* Marshall 1922: 59, by original designation.

#### Diagnosis and description.

Girón and Franz (2010) present a detailed diagnosis and description of the genus. Their diagnosis (slightly modified) follows here: *Apodrosus* is a genus of relatively small sized (2-7 mm), often metallic colored (but not among the Cuban species), exclusively Caribbean entimine weevils without a pronotal postocular lobe and postocular vibrissae, and with the humeri and wings well developed. Species of *Apodrosus* may resemble members of the Anypotactini and Polydrusini; however, *Apodrosus* can be distinguished from *Polydrusus* and other polydrusine genera by a particular combination of characters including a median furrow on the head, a large, bare, and smooth triangular area formed by the epistome on the rostrum; the presence of premucro; the presence of a median fovea on sternum VII; and an either J- or Y-shaped female spermatheca. *Apodrosus* is furthermore distinguished from an undescribed though apparently closely related genus that also occurs at higher elevations in the Hispaniolan Cordillera by having a well deﬁned epistome, well developed elytral humeri, and fully developed wings. Finally, *Apodrosus* diﬀers from *Anypotactus* Schoenherr in having connate (as opposed to free) claws.

#### Distribution.

Species of *Apodrosus* are known from the Bahamas, Cuba, Dominican Republic, Haiti, Mona Island, Puerto Rico, and the Turks and Caicos Islands. The records of the genus from Cuba are reported herein for the first time.

### Description of species

#### Apodrosus alberti

Taxon classificationAnimaliaColeopteraCurculionidae

Anderson sp. n.

http://zoobank.org/4704330D-0DD7-4513-8F72-1D4DE2CDF21C

Figures 1–3

##### Specimens examined.

5 males, 3 females. Holotype male (CMNC), labelled CUBA: Province Granma, Parque Nacional Pico Turquino, 1103 m, 20.0107, -76.8843, IV.2012, CarBio Team, montane forest , CU-03. Paratypes. Alto de Merino, near biological station, 958 m, 5–10.III.2013, 19.9858333, -077.0158333, pluviselva litter, F. Cala Riquelme, A. Deler Hernandez (1 female; CMNC). Parque Nacional Pico Turquino, on the trail up to ca. 0.5 km (by air) from La Platica, 20.0083333, -76.8883333, 920 m, 23–27.VI.2012, A. Deler Hernández & M. Fikacek, sweeping exposed vegetation, secondary e**v**ergreen forest, MF-20 (1 female; CMNC). Province Santiago de Cuba, Parque Nacional Gran Piedra, Gran Piedra and surrounding trails, general collecting, 20.01177, -75.62595, 1200 m, leg. N. Franz, 29.I.2012 (1 male; ASUHIC). Gran Piedra, along trail Museo Isabelica to Gran Sofía, on plants, 20.0010, -75.5940, 800 m, leg N. Franz, 28.I.2012 (1 male, 1 female; ASUHIC). Gran Piedra, nr. Santiago, May 30–31, 1957, M.W. Sanderson (2 males; CWOB, INHS).

##### Diagnosis.

This species is easily distinguished from other Cuban species by larger eyes, elytra with all intervals of equal elevation, elytra with stria 10 continuous throughout length, body with most scales brown or copper in color, and distinctive male genitalia.

##### Description.

*Male.* Body length 3.6–4.2 mm; in dorsal view about 2.3 times longer than greatest width which is at about second third of elytra; dorsal outline in lateral view quite flat. Vestiture composed of pale to dark brown scales, with very small recurved, fine brown setae and a few scattered broader white scales on elytra.. Eyes 1.4 times longer than wide, projected, separated from anterior margin of prothorax by 0.3 times greatest diameter of eye; line of anterior margin of eyes very slightly impressed; shortest distance between eyes (dorsal view) 0.35 times greatest width of pronotum; median furrow linear, narrow and deep, extending from anterior to posterior margin of eyes. Rostrum slightly longer than wide; epistoma apically with three setae situated on each side; nasal plate well defined, v-shaped, hind margin tumid, not declivious, continuing onto rostrum as an elevated median carina. Antennal insertion apicad of midpoint of rostrum; scrobe curved downwards by 45°, directed posteriorly at end, barely reaching anterior margin of eye, separated from it by 2.5 times width of scrobe. Mandibles with 2 lateral setae. Antennae reddish brown; antennal scape extending to just slightly before posterior margin of eye; desmomere I about same length as II. Pronotum cylindrical, slightly wider than long, greatest width from midlength to near base; dorsal surface shallowly punctate, scales sparse, each puncture with a curved, fine brown seta; posterior margin slightly bisinuate, slightly wider than anterior margin; scutellum subcircular, glabrous. Mesocoxal cavities about 5 times width of intercoxal process. Metasternum with lateral portions tumid, not posteriorly produced. Elytra in dorsal view 1.75 times their greatest width; anterior margin sinuate; humeral region of elytra 1.5 times width of posterior margin of pronotum; lateral margins slightly divergent until second third, thereafter convergent; apex acutely rounded; in lateral view with dorsal outline quite flat; posterior declivity gradually descending; striae 9 and 10 complete throughout length although punctures of 10 faintly defined beyond metacoxa; intervals largely covered with scales, with dark and light areas forming an irregular pattern; all intervals equally flat, humerus angled; interval 9 very slightly tumid just anterior to metacoxa; all intervals with recurved, fine brown setae, a few scattered broader white scales on elytral declivity. Venter with scales sparse, small on ventrites, middle of ventrites 1 and 2 with moderately dense, long, fine, erect hairs, ventrites 3 and 4 subequal in length, their combined length slightly longer than ventrite 5; posterior margin of ventrite 5 widely rounded, apex at middle narrowly impressed. Tergum VII of male emarginate. Tegmen with tegminal apodeme 0.5 times length of aedeagus; tegminal plate simple. Aedeagus in dorsal view about 5 times longer than its greatest width; apex rounded. Endophallus extended to about midlength of aedeagal apodemes, with only a pair of inverted c-shaped sclerites at about one-third length of aedeagus. Aedeagus in lateral view slightly evenly convex. Aedeagal apodemes about 0.8 times length of aedeagus.

*Female*. Body length 3.9–4.0 mm. Differing from male as follows: elytra in lateral view with posterior declivity angulate, sutural interval very slightly inflated at about midheight declivity.

##### Etymology.

This species is named after Albert Deler Hernández, coleopterist, of Santiago de Cuba, Cuba.

##### Natural history.

Adults were collected on vegetation along trails in montane forest.

##### Comments.

Dissected males from Parque Nacional Pico Turquino and Parque Nacional Gran Piedra have indistinguishable genitalia and are here considered conspecific.

**Figures 1–3.**
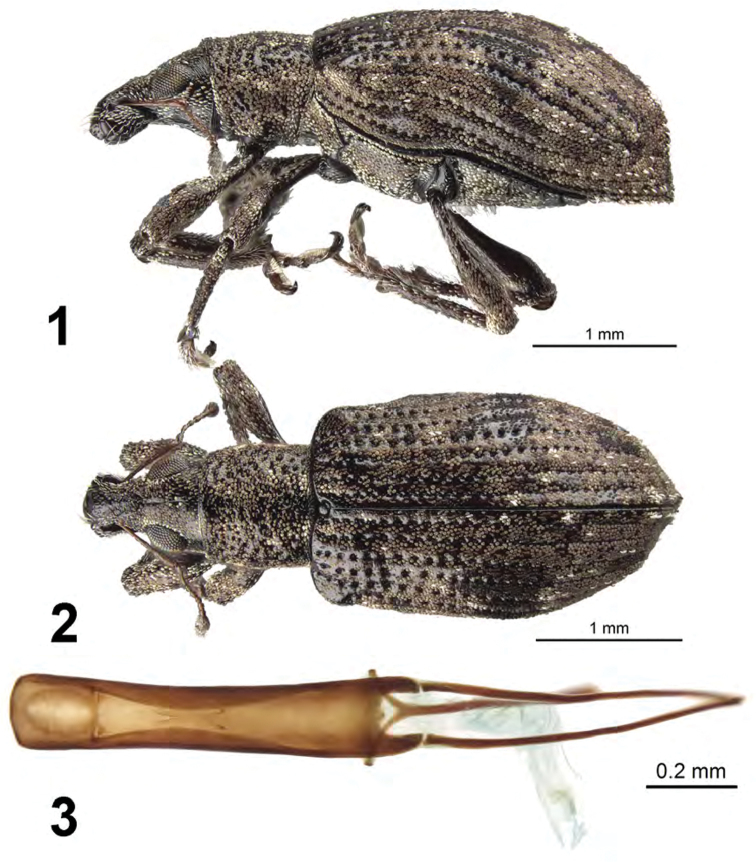
*Apodrosus alberti*. **1** Lateral habitus, female **2** Dorsal habitus, female **3** Male aedeagus, dorsal.

#### Apodrosus alternatus

Taxon classificationAnimaliaColeopteraCurculionidae

Anderson sp. n.

http://zoobank.org/11001A82-F052-46CB-8835-8EB117681F96

Figures 4–6

##### Specimens examined.

5 males, 8 females. Holotype male (CMNC), labelled CUBA: Province Guantanamo, El Yunque, 360 m, 20.34501, -74.56642, IV.2014, CarBio Team, CU-15. Paratypes. Same data as holotype (1 male, 2 females; CMNC). El Yunque, 20–150 m, 20.317, -74.571, 31.I.2012, R. Anderson, wet rainforest (1 male; CMNC). El Yunque, along trail to peak, 20.35060, -74.57497, 550 m, beating shrubs and trees, leg. N. Franz, 1.II.2012 (4 females; ASUHIC). El Yunque, Finca La Delicia, 4 km SW Flora y Fauna Station, 20.32085, -74.57014, 150 m, F. Cala, 1.II.2012 (1 female; ASUHIC). El Yunque, along road near Flora y Fauna Station, 20.32775, -74.56941, 100 m, N. Franz, 31.I.2012 (2 males; ASUHIC). Nibujón, Parque Nacional Humboldt, Sendero Mirador, 3 km N. Nibujón, 20.52036, -74.69018, 100 m, on plants, N. Franz, 2.II.2012 (1 female; ASUHIC).

##### Diagnosis.

This species is distinguished from other Cuban species by larger eyes, elytra with intervals 3 (subbasally and discally), 5 (subbasally) and 7 (at humerus) elevated above adjacent intervals (moreso in female than in male), and distinctive male genitalia with the aedeagus length about 4.5 times maximum width.

##### Description.

*Male.* Body length 3.0–3.4 mm; in dorsal view 2.1–2.3 times longer than greatest width which is between midlength and second third of elytra; dorsal outline in lateral view slightly convex. Vestiture composed of pink, pinkish-white to brown scales, with very small recurved, fine brown setae. Eyes 1.6 times longer than wide, projected, separated from anterior margin of prothorax by 0.25 times greatest diameter of eye; line of anterior margin of eyes slightly impressed; shortest distance between eyes (dorsal view) 0.3–0.4 times greatest width of pronotum; median furrow linear, narrow and deep, extending from anterior margin of eye but not reaching anterior margin of pronotum. Rostrum slightly longer than wide; epistoma apically with a single seta situated on each side; nasal plate well defined, tumid, steeply declivious. Antennal insertion apicad of midpoint of rostrum; scrobe curved downwards by 45°, directed posteriorly at end, barely reaching anterior margin of eye, separated from it by 1.5 times width of scrobe. Mandibles with 2 lateral setae. Antennae reddish brown; antennal scape extending to just slightly before posterior margin of eyes; desmomere I about same length as II. Pronotum cylindrical, about as long as wide, greatest width near base; dorsal surface shallowly punctate, each puncture with a curved, fine brown seta; posterior margin slightly bisinuate, slightly wider than anterior margin; scutellum subcircular, rugose and glabrous. Mesocoxal cavities about 3 times width of intercoxal process. Metasternum with lateral portions posteriorly produced. Elytra in dorsal view 1.5 times their greatest width; anterior margin slightly sinuate; humeral region of elytra 1.5 times width of posterior margin of pronotum; lateral margins slightly divergent until second third, thereafter convergent; apex acutely rounded; in lateral view with dorsal outline slightly convex; posterior declivity gradually descending; striae 9 and 10 separate along entire length; intervals completely covered with scales, with dark and light areas forming an irregular pattern; interval 3 slightly produced at base and again at middle on disc, interval 4 tumid at base, interval 5 produced at base, then less so, then pronounced again throughout most of length, humerus distinctly angled; interval 9 slightly tumid above metacoxa; all intervals with recurved, fine brown setae. Venter with scales denser, larger on ventrites 1 and 2, small and fine, some seta-like, on ventrites 3–5; ventrite 2 about as long as ventrite 1 (medially), ventrites 3 and 4 subequal in length, their combined length slightly shorter than ventrite 5; posterior margin of ventrite 5 widely rounded, finely narrowly emarginate at middle, apex at middle narrowly impressed. Tegmen with tegminal apodeme 0.6 times length of aedeagus; tegminal plate simple. Aedeagus in dorsal view about 4.5 times longer than its greatest width; apex rounded. Endophallus with a pair of large asymmetrical hook-like sclerites positioned near midlength, two elongate fields of microtrichiae extended between aedeagal apodemes, and a faint apical sclerite complex. Aedeagus in lateral view slightly evenly convex. Aedeagal apodemes about 0.5 times length of aedeagus.

*Female*. Body length 3.3–3.8 mm. Differing from male as follows: elytra with interval 3 produced at base and again more strongly so at middle on disc, interval 4 tumid at base, interval 5 strongly produced at base, then less so, then moderately pronounced again throughout most of length, humerus distinctly angled, humeral interval somewhat produced a short distance beyond humerus.

##### Etymology.

This species is named after the Latin adjective “alternatus”, referring to the alternating elevation of the elytral intervals.

##### Natural history.

Adults were collected beating vegetation along trails in tropical wet forest.

##### Comments.

During the initial phase of this study, this species and *A. pseudoalternatus* were considered as conspecific. Males can be separated by the form of the aedeagus but females at present are not separable using external features.

**Figures 4–6.**
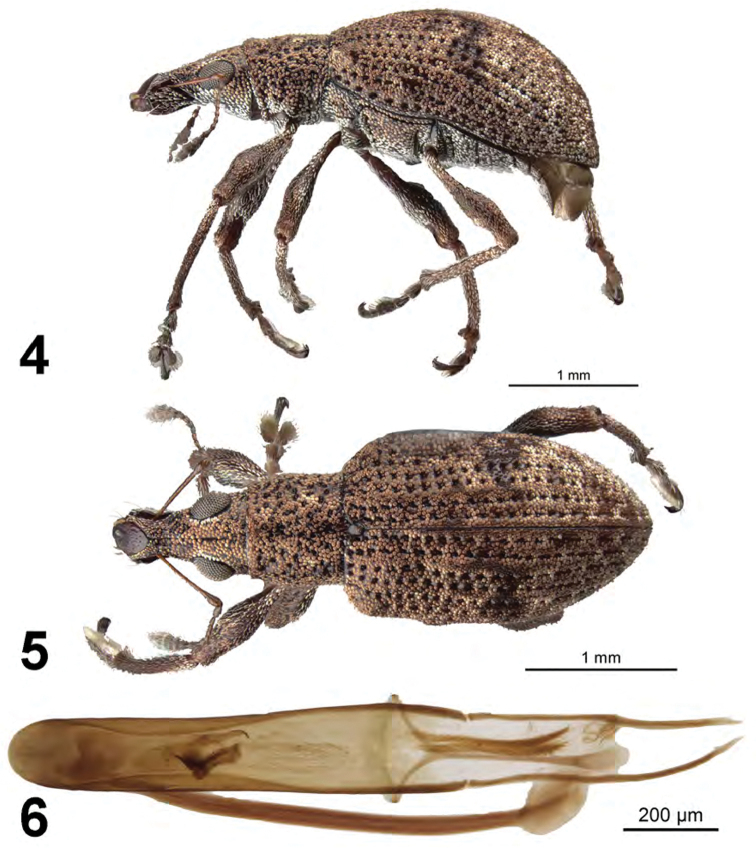
*Apodrosus alternatus*. **4** Lateral habitus, male **5** Dorsal habitus, male **6** Male aedeagus, dorsal.

#### Apodrosus beckeli

Taxon classificationAnimaliaColeopteraCurculionidae

Anderson sp. n.

http://zoobank.org/CF91E267-40E2-4BFC-BA5F-BB6E503D1269

Figures 7–9

##### Specimens examined.

4 males, 10 females. Holotype male (CMNC), labelled CUBA: Province Guantánamo, 8 km W. Imías, 27 m, 20.05421, -74.71520, 4.x.2014, R. Anderson, F. Cala Riquelme, A. Deler Hernandez, 2014-034, beating, coastal scrub. Paratypes. Data as holotype (2 males, 9 females; ASUHIC, CMNC, CWOB). Baracoa, Aug. [18]90, Busch collector (1 male, 1 female; USNM).

##### Diagnosis.

This species is difficult to distinguish from other Cuban species especially *A. franklyni* and *A. griseus*. This group of three species can be separated from other Cuban species by larger eyes, elytra with all intervals of equal elevation, and elytra with stria 10 interrupted above metacoxa. Males of the three species can be sepaarted on the basis of distinctive male genitalia. Females of this species can be separated from *A. franklyni* by the form of the elytral declivity in lateral view but are not separable from *A. griseus* using external features.

##### Description.

*Male.* Body length 2.2–2.3 mm; in dorsal view about 2.2 times longer than greatest width which is at about second third of elytra; dorsal outline in lateral view quite flat. Vestiture composed of grey, greyish-white to brown scales, with very small recurved, fine brown setae. Eyes 1.3 times longer than wide, projected, separated from anterior margin of prothorax by 0.6 times greatest diameter of eye; line of anterior margin of eyes very slightly impressed; shortest distance between eyes (dorsal view) 0.5 times greatest width of pronotum; median furrow linear, narrow and shallow, extending from anterior margin of eyes but not reaching anterior margin of pronotum. Rostrum slightly longer than wide; epistoma apically with three setae situated on each side; nasal plate well defined, v-shaped, slightly tumid, not declivious. Antennal insertion apicad of midpoint of rostrum; scrobe curved downwards by 60°, directed posteriorly at end, barely reaching anterior margin of eye, separated from it by 1.5 times width of scrobe. Mandibles with 2 lateral setae. Antennae reddish brown; antennal scape extending to just slightly before posterior margin of eye; desmomere I about same length as II. Pronotum cylindrical, slightly wider than long, greatest width from midlength to near base; dorsal surface shallowly punctate but largely obscured by scales, each puncture with a curved, fine brown seta; posterior margin slightly bisinuate, slightly wider than anterior margin; scutellum subcircular, glabrous. Mesocoxal cavities about 3 times width of intercoxal process. Metasternum with lateral portions slightly tumid, not posteriorly produced. Elytra in dorsal view 1.7–1.8 times their greatest width; anterior margin sinuate; humeral region of elytra 1.5 times width of posterior margin of pronotum; lateral margins subparallel until second third, thereafter convergent; apex acutely rounded; in lateral view with dorsal outline quite flat; posterior declivity gradually descending; stria 9 complete, stria 10 interrupted above metacoxa, resuming at suture between ventrites 1 and 2; intervals completely covered with scales, with dark and light areas forming an irregular pattern; all intervals equally flat, humerus angled; interval 9 very slightly tumid above metacoxa; all intervals with recurved, fine brown setae. Venter with scales dense, large on ventrites; ventrites 3 and 4 subequal in length, their combined length slightly shorter than ventrite 5; posterior margin of ventrite 5 widely rounded, apex at middle narrowly impressed. Tegmen with tegminal apodeme 0.5 times length of aedeagus; tegminal plate simple. Aedeagus in dorsal view about 4.5 times longer than its greatest width; apex rounded. Endophallus extended to about midlength of aedeagal apodemes, with only an apical hooked sclerite complex. Aedeagus in lateral view slightly evenly convex. Aedeagal apodemes about same length as aedeagus.

*Female*. Body length 2.5–3.0 mm.

##### Etymology.

This species is named after William Edwin Beckel, PhD., Entomology, father of Margaret Beckel, President and CEO of the Canadian Museum of Nature, for his generous support of insect taxonomy.

##### Natural history.

Adults were collected beating vegetation in dry coastal scrub.

##### Comments.

Externally, this species is very similar to *A. griseus* and although males can be separated by details in the structure of the endophallus (see key), females cannot be separated using external features.

**Figures 7–9.**
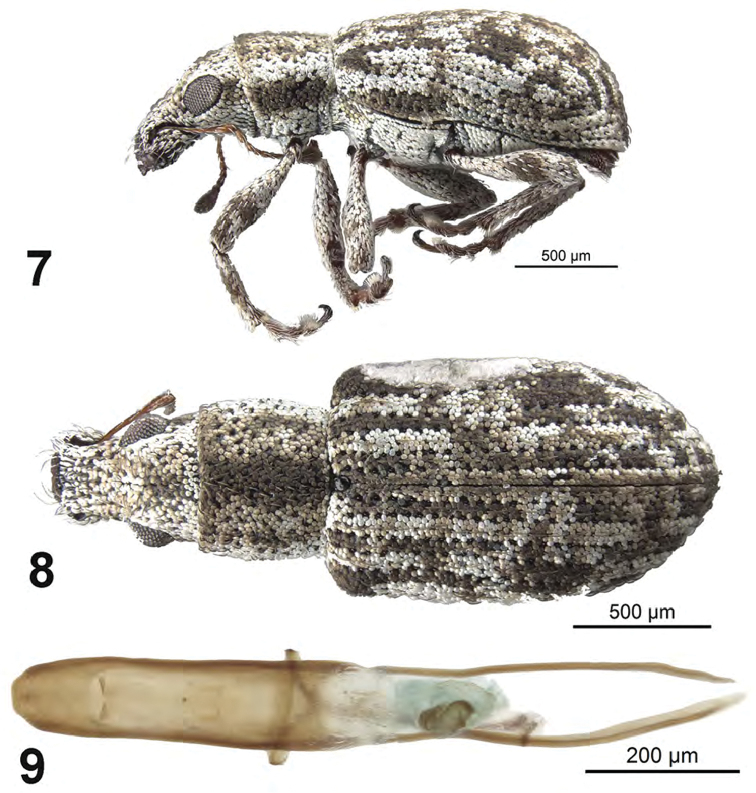
*Apodrosus beckeli*. **7** Lateral habitus, male **8** Dorsal habitus, male **9** Male aedeagus, dorsal.

#### Apodrosus franklyni

Taxon classificationAnimaliaColeopteraCurculionidae

Anderson sp. n.

http://zoobank.org/326B2551-FD08-4A05-A684-AEE7A05091B8

Figures 10–12

##### Specimens examined.

9 males, 4 females. Holotype male (CMNC), labelled CUBA: Province Cienfuegos, Parque Nacional Pico San Juan, road, 21.98812, -80.14632, 1086 m, 19.v.2013, R. Anderson, 2013-022X, hand collections. Paratypes. Data as holotype (5 males, 3 females; CMNC, CWOB). Pico San Juan, near peak, 21.9886833, -80.1465833, 1105 m, 19.V.2013, G. Zhang, CB-13, L.22 (3 males, 1 female; ASUHIC).

##### Diagnosis.

This species is difficult to distinguish from other Cuban species especially *A. beckeli* and *A. griseus*. This group of three species can be separated from other Cuban species by larger eyes, elytra with all intervals of equal elevation, and elytra with stria 10 interrupted above metacoxa. Males of the three species can be separated on the basis of distinctive male genitalia. Females of this species can be separated from *A. beckeli* and *A. griseus* by the elytral profile at apical declivity distinctly angulate.

##### Description.

*Male.* Body length 2.8–3.2 mm; in dorsal view about 2.2 times longer than greatest width which is at about second third of elytra; dorsal outline in lateral view quite flat. Vestiture composed of grey, greyish-white to brown scales, with very small recurved, fine brown setae. Eyes 1.3 times longer than wide, projected, separated from anterior margin of prothorax by 0.5 times greatest diameter of eye; line of anterior margin of eyes very slightly impressed; shortest distance between eyes (dorsal view) 0.6 times greatest width of pronotum; median furrow linear, narrow and shallow, extending from anterior margin of eyes but not reaching anterior margin of pronotum. Rostrum slightly longer than wide; epistoma apically with three setae situated on each side; nasal plate well defined, v-shaped, slightly tumid, not declivious. Antennal insertion apicad of midpoint of rostrum; scrobe curved downwards by 60°, directed posteriorly at end, not reaching anterior margin of eye, separated from it by 2.0 times width of scrobe. Mandibles with 2 lateral setae. Antennae reddish brown; antennal scape extending to slightly beyond midlength of eye; desmomere I slightly longer than II. Pronotum cylindrical, slightly longer than wide, greatest width from midlength to near base; dorsal surface shallowly punctate, each puncture with a curved, fine brown seta; posterior margin slightly bisinuate, slightly wider than anterior margin; scutellum subcircular, glabrous. Mesocoxal cavities about 5 times width of intercoxal process. Metasternum with lateral portions slightly tumid, not posteriorly produced. Elytra in dorsal view 1.4–1.5 times their greatest width; anterior margin sinuate; humeral region of elytra 1.7 times width of posterior margin of pronotum; lateral margins slightly divergent until second third, thereafter convergent; apex acutely rounded; in lateral view with dorsal outline quite flat; posterior declivity gradually descending; stria 9 complete, stria 10 interrupted above metacoxa, resuming at suture between ventrites 1 and 2; intervals completely covered with scales, with dark and light areas forming an irregular pattern although many specimens with a more or less distinct transverse dark macula at about posterior one-third of elytral length; all intervals equally flat, humerus angled; interval 9 very slightly tumid above metacoxa; all intervals with recurved, fine brown setae. Venter with scales dense, large on ventrites, ventrites 3 and 4 subequal in length, their combined length slightly about same length as ventrite 5; posterior margin of ventrite 5 widely rounded, apex at middle narrowly impressed. Tegmen with tegminal apodeme 0.55 times length of aedeagus; tegminal plate simple. Aedeagus in dorsal view about 4.7 times longer than its greatest width; apex rounded. Endophallus extended almost to apex of aedeagal apodemes, with a narrow u-shaped sclerite at midlength aedeagus and apical hooked sclerite complex. Aedeagus in lateral view slightly evenly convex. Aedeagal apodemes slightly shorter than length aedeagus.

*Female*. Body length 3.4–3.6 mm. Differing from male as follows: elytra in lateral view slightly tumid dorsally and with posterior declivity angulate, sutural interval very slightly inflated at about midheight of declivity.

##### Etymology.

This species is named after Franklyn Cala Riquelme, arachnologist, of Santiago de Cuba, Cuba.

##### Natural history.

Adults were collected beating montane vegetation along the upper part of the road to Pico San Juan.

##### Comments.

This species and *A. griseus* and *A. beckeli* are superficially very similar. Males can be separated by the form of genitalia but females are very similar with females of *A. franklyni*, differing in the form of the elytral declivity in lateral view.

**Figures 10–12.**
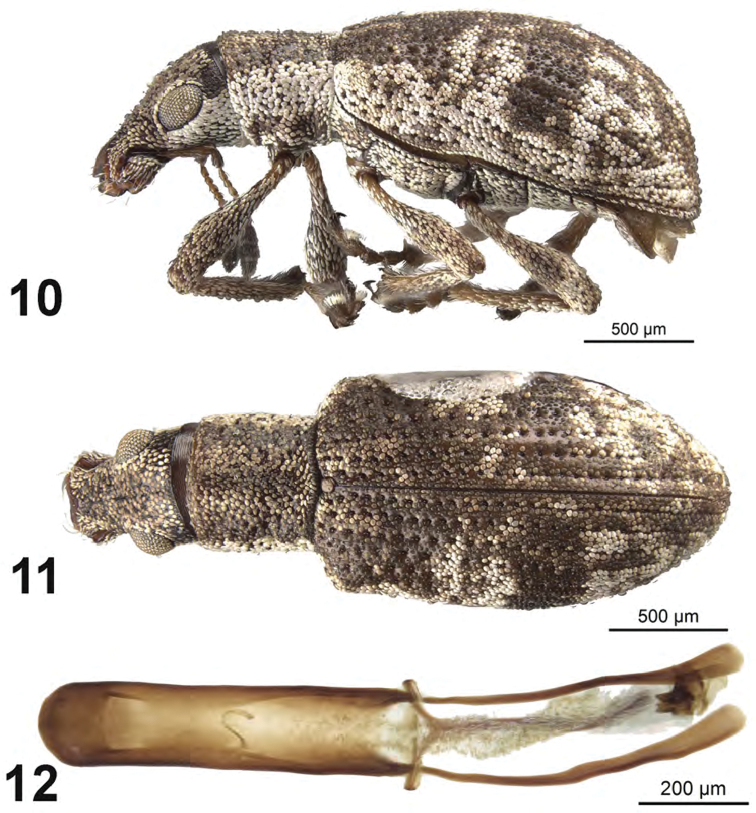
*Apodrosus franklyni*. **10** Lateral habitus, male **11** Dorsal habitus, male **12** Male aedeagus, dorsal.

#### Apodrosus griseus

Taxon classificationAnimaliaColeopteraCurculionidae

Anderson sp. n.

http://zoobank.org/E4B8DAB9-E57A-4536-9313-363A008ECAF4

Figures 13–15

##### Specimens examined.

6 males, 4 females. Holotype male (CMNC), labelled CUBA: Province Santiago de Cuba, Siboney-Jutici Ecological Reserve, 60 m, near Biological Station, 19.96158, -75.71534, 1.IV.2012, CarBio Team, forest semi-dry, broadleaf, CU-07. Paratypes. Siboney-Jutici Ecological Reserve, Estación Ecologica Siboney, 50 m, 19.961, -75.715, 6.II.2012, R. Anderson, dry thorn scrub (2 males, 2 females; CMNC). Siboney-Jutici Ecological Reserve, 19.96227, -75.71684, 100 m, beating shrubs at night, leg. F. Cala, 26.I.2012 (2 males, 1 female; ASUHIC). Siboney-Jutici Ecological Reserve, 19.96227, -75.71684, 100 m, beating shrubs at night, leg. N. Franz, 6.II.2012 (1 male, 1 female; ASUHIC).

##### Diagnosis.

This species is difficult to distinguish from other Cuban species especially *A. franklyni* and *A. beckeli*. This group of three species can be separated from other Cuban species by larger eyes, elytra with all intervals of equal elevation, and elytra with stria 10 interrupted above metacoxa. Males of the three species can be sepaarted on the basis of distinctive male genitalia. Females of this species can be separated from *A. franklyni* by the form of the elytral declivity in lateral view but are not separable from *A. beckeli* using external features.

##### Description.

*Male.* Body length 2.6–2.9 mm; in dorsal view 2.2–2.4 times longer than greatest width which is between first and second third of elytra; dorsal outline in lateral view quite flat. Vestiture composed of grey, greyish-white to brown scales, with very small recurved, fine brown setae. Eyes 1.3 times longer than wide, projected, separated from anterior margin of prothorax by 0.7 times greatest diameter of eye; line of anterior margin of eyes very slightly impressed; shortest distance between eyes (dorsal view) 0.3–0.4 times greatest width of pronotum; median furrow linear, narrow and shallow, extending from anterior margin of eyes but not reaching anterior margin of pronotum. Rostrum slightly longer than wide; epistoma apically with three setae situated on each side; nasal plate well defined, v-shaped, slightly tumid, not declivious. Antennal insertion apicad of midpoint of rostrum; scrobe curved downwards by 60°, directed posteriorly at end, barely reaching anterior margin of eye, separated from it by 1.5 times width of scrobe. Mandibles with 2 lateral setae. Antennae reddish brown; antennal scape extending to just slightly before posterior margin of eye; desmomere I very slightly longer than II. Pronotum cylindrical, slightly wider than long, greatest width near base; dorsal surface shallowly punctate but largely obscured by scales, each puncture with a curved, fine brown seta; posterior margin slightly bisinuate, slightly wider than anterior margin; scutellum subcircular, glabrous. Mesocoxal cavities about 3 times width of intercoxal process. Metasternum with lateral portions slightly tumid, not posteriorly produced. Elytra in dorsal view 1.7–1.8 times their greatest width; anterior margin sinuate; humeral region of elytra 1.5 times width of posterior margin of pronotum; lateral margins subparallel until second third, thereafter convergent; apex acutely rounded; in lateral view with dorsal outline quite flat; posterior declivity gradually descending; stria 9 complete, stria 10 interrupted above metacoxa, resuming at suture between ventrites 1 and 2; intervals completely covered with scales, with dark and light areas forming an irregular pattern; all intervals equally flat, humerus angled; interval 9 very slightly tumid above metacoxa; all intervals with recurved, fine brown setae. Venter with scales dense, large laterally on ventrites, smaller and less dense medially, ventrites 3 and 4 subequal in length, their combined length slightly shorter than ventrite 5; posterior margin of ventrite 5 widely rounded, apex at middle narrowly impressed. Tegmen with tegminal apodeme 0.8 times length of aedeagus; tegminal plate simple. Aedeagus in dorsal view about 6.5 times longer than its greatest width; apex rounded. Endophallus extended to apical two-thirds of aedeagal apodemes, with a narrow, scythe-like sclerite positioned near basal two-thirds of length, an elongate field of microtrichia positioned at base of aedeagus, and an apical hooked sclerite complex. Aedeagus in lateral view slightly evenly convex. Aedeagal apodemes about one-half length aedeagus.

*Female*. Body length 3.3–3.8 mm.

##### Etymology.

This species is named after the Latin adjective “griseus” meaning grey, after the predominantly grey scales of the body vestiture.

##### Natural history.

Adults were collected beating vegetation in dry thorn scrub.

##### Comments.

Externally, this species is very similar to *A. beckeli* and although males can be separated by details in the structure of the endophallus (see key), females cannot be separated using external features.

**Figures 13–15.**
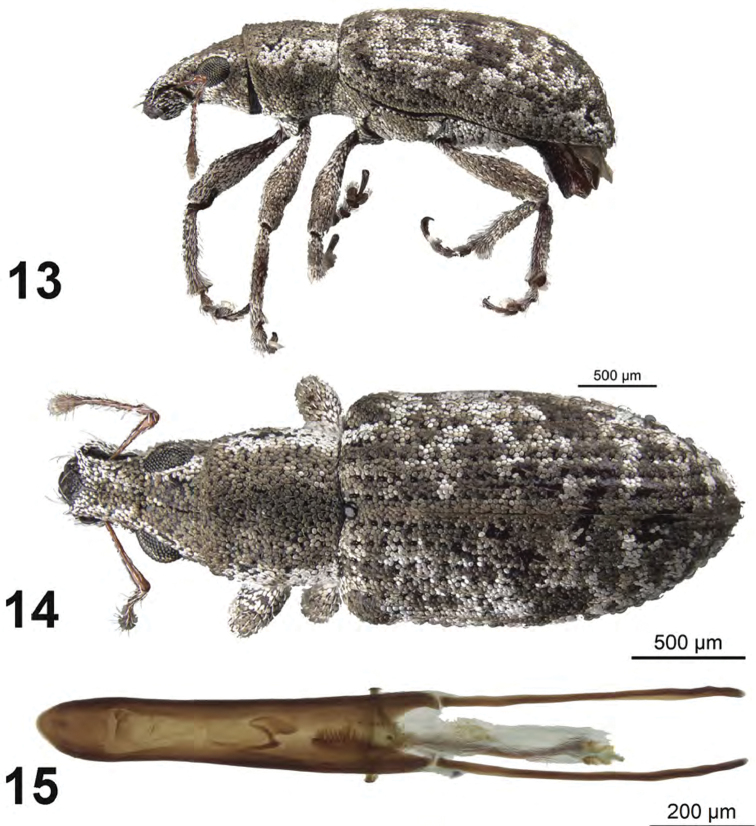
*Apodrosus griseus*. **13** Lateral habitus, male **14** Dorsal habitus, male **15** Male aedeagus, dorsal.

#### Apodrosus mensurensis

Taxon classificationAnimaliaColeopteraCurculionidae

Anderson sp. n.

http://zoobank.org/417AA45B-F711-4A1C-92BE-9259930BB9EB

Figures 16–18

##### Specimens examined.

2 males. Holotype male (ASUHIC), labelled CUBA: Province Holguin, Mayari, Parque Nacional La Mensura-Piloto, 20.5288333, -75.7683000, 750 m, 09 May 2013, G. Zhang, (CB13_L2). Paratype. Data as holotype (1 male; CMNC).

##### Diagnosis.

This species is distinguished from other Cuban species by larger eyes, elytra with all intervals of equal elevation, elytra with stria 10 continuous throughout length, body with most scales brown or copper in color, and distinctive male genitalia.

##### Description.

*Male.* Body length 2.7–3.0 mm; in dorsal view about 2.2 times longer than greatest width which is at about second third of elytra; dorsal outline in lateral view quite flat. Vestiture composed of predominantly light to dark brown scales, with very small recurved, fine dark brown setae. Eyes 1.3 times longer than wide, projected, separated from anterior margin of prothorax by 0.6 times greatest diameter of eye; line of anterior margin of eyes very slightly impressed; shortest distance between eyes (dorsal view) 0.4 times greatest width of pronotum; median furrow linear, narrow and shallow, extending from just behind anterior margin of eyes, not reaching anterior margin of pronotum. Rostrum slightly longer than wide; epistoma apically with four setae situated on each side; nasal plate well defined, v-shaped, slightly tumid, slightly declivious. Antennal insertion apicad of midpoint of rostrum; scrobe curved downwards by about 45°, directed posteriorly towards apex, not reaching anterior margin of eye, separated from it by about width of scrobe. Mandibles with 2 lateral setae. Antennae reddish brown; antennal scape extending to just slightly before posterior margin of eye; desmomere I slightly longer than II. Pronotum cylindrical, very slightly wider than long, greatest width at about midlength; dorsal surface shallowly punctate but largely obscured by scales, each puncture with a curved, fine brown seta; posterior margin slightly bisinuate, about same width as anterior margin; scutellum subcircular, glabrous. Mesocoxal cavities about 6 times width of intercoxal process. Metasternum with lateral portions slightly tumid, not posteriorly produced. Elytra in dorsal view 1.5–1.6 times their greatest width; anterior margin sinuate; humeral region of elytra 1.5 times width of posterior margin of pronotum; lateral margins subparallel until second third, thereafter convergent; apex acutely rounded; in lateral view with dorsal outline quite flat; posterior declivity gradually descending; striae 9 and 10 complete; intervals completely covered with scales, with dark and light areas forming an irregular pattern but with declivity mainly of paler scales; all intervals equally flat, humerus angled; interval 9 very slightly tumid above metacoxa; all intervals with recurved, fine brown setae. Venter with scales dense, large on ventrites, ventrites 3 and 4 subequal in length, their combined length slightly shorter than ventrite 5; posterior margin of ventrite 5 widely rounded, finely narrowly emarginate at middle, apex at middle deeply narrowly impressed. Tegmen with tegminal apodeme 0.5 times length of aedeagus; tegminal plate simple. Aedeagus in dorsal view about 4.5 times longer than its greatest width; tapered in apical 2/5 to narrowly rounded apex. Endophallus extended to about midlength of aedeagal apodemes, with only pair of small curved sclerites just before midlength, and a pair of narrow, faint fields of microtrichiae near base aedeagus. Aedeagus in lateral view slightly evenly convex. Aedeagal apodemes about same length as aedeagus.

*Female*. Unknown.

##### Etymology.

This species is named after the type locality Parque Nacional La Mensura-Piloto. The specific name is an adjective.

##### Natural history.

Adults were collected beating vegetation in a mixed pine scrub.

**Figures 16–18.**
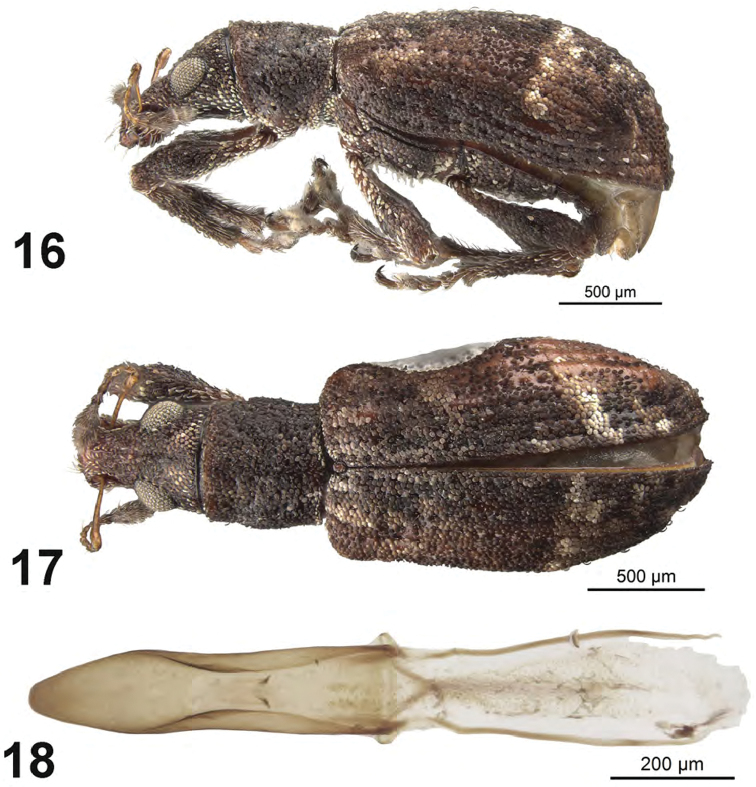
*Apodrosus mensurensis*. **16** Lateral habitus, male **17** Dorsal habitus, male **18** Male aedeagus, dorsal.

#### Apodrosus pseudoalternatus

Taxon classificationAnimaliaColeopteraCurculionidae

Anderson sp. n.

http://zoobank.org/621091D2-ED04-4D2A-97DA-647D353DF260

Figures 19–21

##### Specimens examined.

1 male, 1 female. Holotype male (CMNC), labelled CUBA: Province Matanzas, Varadero, Varahicacos, 8 m, 23.194, -81.154, III.2014, F. Cala Riquelme. Paratype. Same data as holotype (1 female; CMNC).

##### Diagnosis.

This species is distinguished from other Cuban species by larger eyes, elytra with intervals 3 (subbasally and discally), 5 (subbasally) and 7 (at humerus) elevated above adjacent intervals (more so in female than in male), and distinctive male genitalia with the aedeagus very long and slender, length about 10 times maximum width.

##### Description.

*Male.* Body length 3.0 mm; in dorsal view 2.3 times longer than greatest width which is between midlength and second third of elytra; dorsal outline in lateral view moderately convex. Vestiture composed of pink, pinkish-white to brown scales, with very small recurved, fine brown setae. Eyes 1.6 times longer than wide, projected, separated from anterior margin of prothorax by 0.25 times greatest diameter of eye; line of anterior margin of eyes slightly impressed; shortest distance between eyes (dorsal view) 0.4 times greatest width of pronotum; median furrow linear, narrow and deep, extending from anterior margin of eye but not reaching anterior margin of pronotum. Rostrum slightly longer than wide; epistoma apically with single setasituated on each side; nasal plate well defined, tumid, steeply declivious. Antennal insertion apicad of midpoint of rostrum; scrobe curved downwards by 45°, directed posteriorly at end, barely reaching anterior margin of eye, separated from it by 1.5 times width of scrobe. Mandibles with 2 lateral setae. Antennae reddish brown; antennal scape extending to just slightly before posterior margin of eyes; desmomere I about same length as II. Pronotum cylindrical, about as long as wide, greatest width near base; dorsal surface shallowly punctate, each puncture with a curved, fine brown seta; posterior margin slightly bisinuate, slightly wider than anterior margin; scutellum subcircular, rugose and glabrous. Mesocoxal cavities about 3 times width of intercoxal process. Metasternum with lateral portions posteriorly produced. Elytra in dorsal view 1.5 times their greatest width; anterior margin sinuate; humeral region of elytra 1.5 times width of posterior margin of pronotum; lateral margins slightly divergent until second third, thereafter convergent; apex acutely rounded; in lateral view with dorsal outline slightly convex; posterior declivity gradually descending; striae 9 and 10 separate along entire length; intervals completely covered with scales, with dark and light areas forming an irregular pattern; interval 3 slightly produced at base and again at middle on disc, interval 4 tumid at base, interval 5 produced at base, then less so, then pronounced again throughout most of length, humerus distinctly angled; interval 9 slightly tumid above metacoxa; all intervals with recurved, fine brown setae. Venter with scales denser, larger on ventrites 1 and 2, small and fine, some seta-like, on ventrites 3-5; ventrite 2 about as long as ventrite 1 (medially), ventrites 3 and 4 subequal in length, their combined length slightly shorter than ventrite 5; posterior margin of ventrite 5 widely rounded, finely narrowly emarginate at middle, apex at middle narrowly impressed. Tegmen with tegminal apodeme 0.3 times length of aedeagus; tegminal plate simple. Aedeagus in dorsal view about 10 times longer than its greatest width; apex rounded. Endophallus with a pair of asymmetrical hook-like sclerites positioned near midlength otherwise no distinct internal sclerotization. Aedeagus in lateral view slightly evenly convex. Aedeagal apodemes about 0.4 times length of aedeagus.

*Female*. Body length 3.5 mm. Differing from male as follows: elytra with interval 3 produced at base and again more strongly so at middle on disc, interval 4 tumid at base, interval 5 strongly produced at base, then less so, then moderately pronounced again throughout most of length, humerus distinctly angled, humeral interval somewhat produced a short distance beyond humerus.

##### Etymology.

This species is named because of the initial confusion of this species with *A. alternatus*. The specific name is an adjective.

##### Natural history.

No information.

##### Comments.

During the initial phase of this study this species and *A. alternatus* were placed as conspecific. Males can be separated by the form of the aedeagus but females at present are not separable using external features.

**Figures 19–21.**
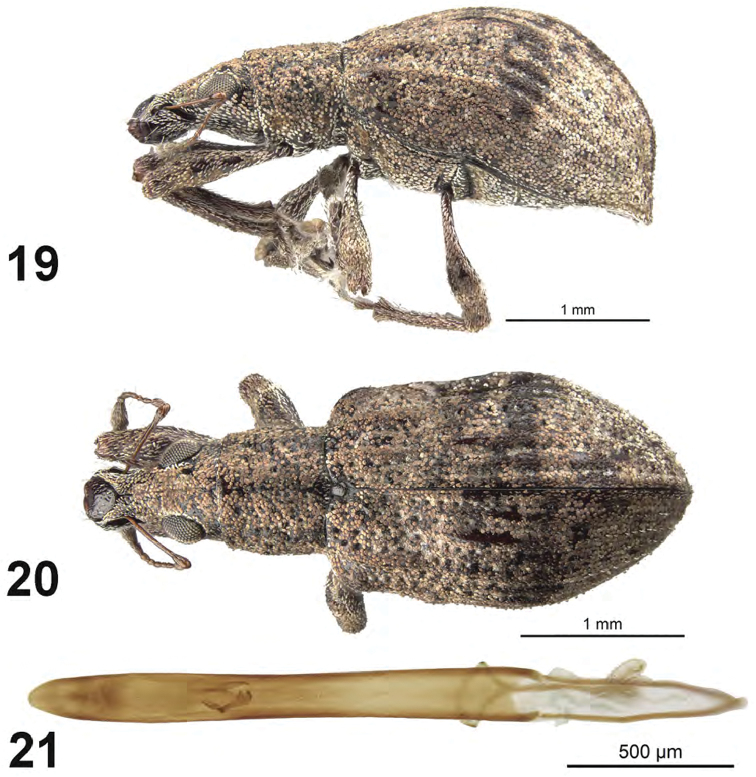
*Apodrosus pseudoalternatus*. **19** Lateral habitus, female **20** Dorsal habitus, female **21** Male aedeagus, dorsal.

#### Apodrosus sandersoni

Taxon classificationAnimaliaColeopteraCurculionidae

Anderson sp. n.

http://zoobank.org/38EB6069-43D2-4BB5-A6EF-A3FB2252E63A

Figures 22–23

##### Specimens examined.

2 females. Holotype female (CWOB), labelled CUBA: Province Oriente, Loma Lafarola, along Ríoo Cajobado, 2.VI.1959, M.W. Sanderson, C59-12. Paratype. Data as holotype (1 female; CMNC).

##### Diagnosis.

This species is distinguished from other Cuban species by larger eyes, elytra with all intervals of equal elevation, elytra with stria 10 continuous throughout length, body with most scales scales grey, greyish white or pearlescent in color. Only females are known.

##### Description.

*Female.* Body length 3.2–3.3 mm; in dorsal view 2.3 times longer than greatest width which is at about second third of elytra; dorsal outline in lateral view slightly convex. Vestiture composed of pale grey to pearlescent scales, with very small recurved, fine greyish setae. Eyes 1.3 times longer than wide, quite flat, separated from anterior margin of prothorax by 0.5 times greatest diameter of eye; line of anterior margin of eyes not at all impressed, frons continuous with base of rostrum; shortest distance between eyes (dorsal view) 0.45 times greatest width of pronotum; median furrow linear, narrow and deep, confined to area between eyes. Rostrum slightly longer than wide; epistoma apically with three setae situated on each side; nasal plate well defined, glabrous, not declivious, continuing onto rostrum as a number of indistinct, small striae, not carinate posteriorly. Antennal insertion apicad of midpoint of rostrum; scrobe curved downwards by 60°, directed posteriorly at end, barely reaching anterior margin of eye, separated from it by about width of scrobe. Mandibles with 2 lateral setae. Antennae reddish brown; antennal scape extending to just slightly before posterior margin of eye; desmomere I very slightly longer than II. Pronotum cylindrical, slightly wider than long, greatest width from midlength to base; dorsal surface shallowly punctate, scales moderately dense, each puncture with a suberect, fine grey seta; posterior margin slightly bisinuate, slightly wider than anterior margin; scutellum subcircular, with a few scattered scales. Mesocoxal cavities about 5 times width of intercoxal process. Metasternum with lateral portions tumid, not posteriorly produced. Elytra in dorsal view 1.6 times their greatest width; anterior margin sinuate; humeral region of elytra 1.5 times width of posterior margin of pronotum; lateral margins slightly divergent until second third, thereafter convergent; apex acutely rounded; in lateral view with dorsal outline slightly convex; posterior declivity gradually descending; striae 9 and 10 complete throughout their length although punctures of 10 faintly defined beyond metacoxa; intervals largely covered with uniformly grey to pearlescent scales; all intervals equally flat, humerus angled; interval 9 very slightly tumid just anterior to metacoxa; all intervals with minuet fine grey setae. Venter with scales scattered, small on ventrites; ventrites 3 and 4 subequal in length, their combined length slightly shorter than ventrite 5; ventrite 5 setose, posterior margin widely rounded, impressed and very narrowly emarginated medially. Female not dissected.

*Male*. Unknown.

##### Etymology.

This species is named after Milton W. Sanderson (1910-2012), entomologist and botanist, Illinois Natural History Survey, Champaign, Illinois.

##### Natural history.

No information.

##### Comments.

This species bears a superficial resemblance to *A. argentatus* from the Dominican Republic and Puerto Rico and can be separated by geographic distribution and the differently sculptured dorsal surface of the rostrum. Males are not available of *A. sandersoni* for comparison of genitalia. We have found a latitude and longitude for Loma La Farola in Guantánamo Province as 20.1592 -74.4686 and we believe these to be the approximate coordinates of specimen capture.

**Figures 22–23.**
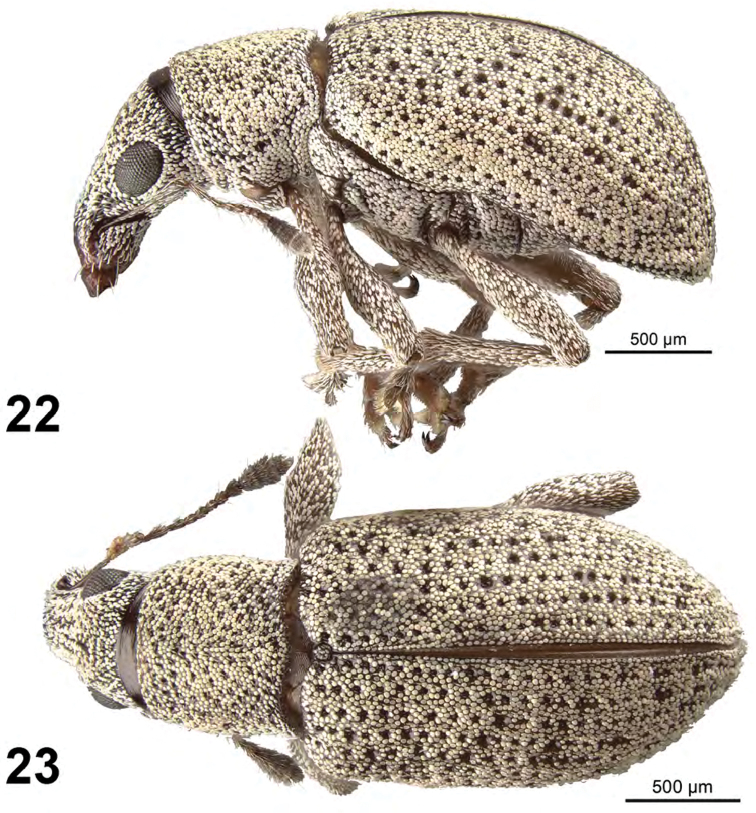
*Apodrosus sandersoni*. **22** Lateral habitus, female **23** Dorsal habitus, female.

#### Apodrosus zayasi

Taxon classificationAnimaliaColeopteraCurculionidae

Anderson sp. n.

http://zoobank.org/3C5872C4-1EE5-4262-B986-FD5D1F7A4785

Figures 24–26

##### Specimens examined.

1 male, 2 females. Holotype male (CMNC), labelled CUBA: Province Cienfuegos, Parque Nacional Pico San Juan, road, 21.98812, -80.14632, 1086 m, 19.V.2013, R. Anderson, 2013-022X, hand collections. Paratype. Data as holotype (1 female; CMNC). Pico San Juan, near peak, 21.9886833, -80.1465833, 1105 m, 19.V.2013, G. Zhang, CB-13, L.22 (1 female; ASUHIC).

##### Diagnosis.

This species is distinguished from other Cuban species by the eyes small, rounded, the distance from posterior margin of eye to posterior margin of head about the same as greatest diameter of an eye, and by distinctive male genitalia. It is the only Cuban species with such small, rounded eyes.

##### Description.

*Male.* Body length 3.6 mm; in dorsal view about 2.3 times longer than greatest width which is at about second third of elytra; dorsal outline in lateral view quite tumid. Vestiture composed of pale to dark brown scales, with very small recurved, fine brown setae. Eyes 1.1 times longer than wide, projected, separated from anterior margin of prothorax by about greatest diameter of eye; line of anterior margin of eyes very slightly impressed; shortest distance between eyes (dorsal view) 0.5 times greatest width of pronotum; median furrow linear, narrow and shallow, extending from anterior margin of eyes but not reaching anterior margin of pronotum, partially obscured by scales. Rostrum slightly longer than wide; epistoma apically with two setae situated on each side; nasal plate well defined, v-shaped, slightly tumid, not declivious. Antennal insertion apicad of midpoint of rostrum; scrobe curved downwards by 45°, directed posteriorly at end, barely reaching anterior margin of eye, separated from it by 2.0 times width of scrobe. Mandibles with 2 lateral setae. Antennae reddish brown; antennal scape extending to just slightly beyond posterior margin of eye; desmomere I about same length as II. Pronotum cylindrical, slightly wider than long, greatest width at midlength; dorsal surface shallowly punctate but largely obscured by scales, each puncture with a curved, fine brown seta; posterior margin slightly bisinuate, slightly wider than anterior margin; scutellum subcircular, glabrous. Mesocoxal cavities about 5 times width of intercoxal process. Metasternum with lateral portions slightly tumid, not posteriorly produced. Elytra in dorsal view 1.8 times their greatest width; anterior margin sinuate; humeral region of elytra 1.5 times width of posterior margin of pronotum; lateral margins slightly divergent until second third, thereafter convergent; apex acutely rounded; in lateral view with dorsal outline tumid; posterior declivity gradually descending; stria 9 complete, stria 10 interrupted above metacoxa (appearing to merge with stria 9), resuming at suture between ventrites 1 and 2; intervals completely covered with scales, with dark and light areas forming an irregular pattern; all intervals equally flat, humerus angled; interval 9 very slightly tumid above metacoxa; all intervals with recurved, fine brown setae. Venter with scales very sparse, linear and hair-like on all ventrites; ventrites 3 and 4 subequal in length, their combined length shorter than ventrite 5; posterior margin of ventrite 5 widely rounded, finely narrowly emarginate at middle, apex at middle narrowly impressed. Tegmen with tegminal apodeme 0.8 times length of aedeagus; tegminal plate simple. Aedeagus short and robust, in dorsal view about 3.0 times longer than its greatest width; apex rounded, deflexed ventrally. Endophallus extended to just beyond base of aedeagus, with no visible internal sclerotization. Aedeagus in lateral view slightly evenly convex. Aedeagal apodemes about same length as aedeagus.

*Female*. Body length 3.4–3.6 mm.

##### Etymology.

This species is named after Fernando de Zayas (1912–1983), entomologist, Cuban Academy of Sciences.

**Figures 24–26.**
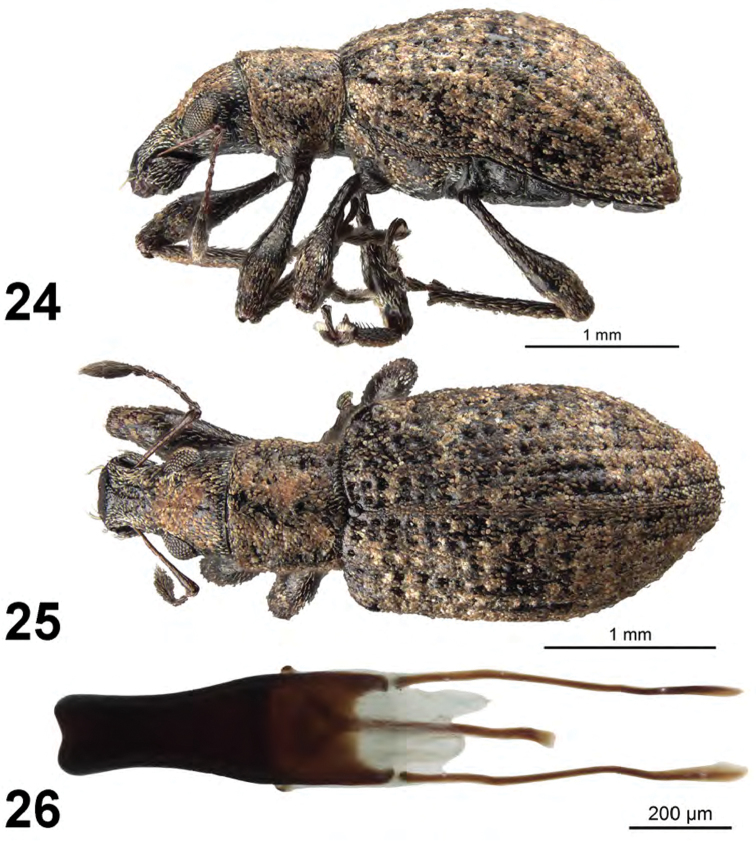
*Apodrosus zayasi*. **24** Lateral habitus, female **25** Dorsal habitus, female **26** Male aedeagus, dorsal.

##### Natural history.

Adults were collected beating vegetation along the upper part of the road to Pico San Juan.

### Key to Cuban species of *Apodrosus*

**Table:** 

1	Eyes small, rounded, the distance from posterior margin of eye to posterior margin of head about the same as greatest diameter of an eye (Fig. 24); male with aedeagus very short and stout, length about 3 times maximum width (Fig. 26)	***A. zayasi* Anderson, sp. n.**
–	Eyes larger, more elongate oval, the distance from posterior margin of eye to posterior margin of head about 0.3-0.7 times greatest diameter of an eye; male with aedeagus with length more than 4 times maximum width	**2**
2	Elytra with intervals 3 (subbasally and discally), 5 (subbasally) and 7 (at humerus) elevated above adjacent intervals (more so in female than in male) (Figs 5, 20)	**3**
–	Elytra with all intervals of equal elevation	**4**
3	Male with aedeagus very long and slender, length about 10 times maximum width (Fig. 21); endophallus lacking any fields of microtrichiae (Fig. 21); females not separable	***A. pseudoalternatus* Anderson, sp. n.**
–	Male with aedeagus moderately long and less slender, length about 4.5 times maximum width (Fig. 6); endophallus with two elongate fields of microtrichiae at base extended between apodemes (Fig. 6); females not separable	***A. alternatus* Anderson, sp. n.**
4	Elytra with stria 10 continuous throughout length (Figs 1, 16, 22)	**5**
–	Elytra with stria 10 interrupted above metacoxa (Figs 7, 13)	**7**
5	Body with most scales grey, greyish white or pearlescent in color (Figs 22, 23); in lateral view with frons and base of rostrum continuous, in same plane (Fig. 22); ventrites 1 and 2 with only scales, not setose; males not known	***A. sandersoni* Anderson, sp. n.**
–	Body with most scales brown or copper in color (Figs 1, 2, 16, 17); frons and base of rostrum separated by a slight transverse impression in lateral view (Figs 1, 16); ventrites 1 and 2 setose	**6**
6	Male with aedeagus in dorsal view broadly rounded at apex, subtruncate (Fig. 3); male tergite VII with apex emarginate; male with apex of ventrite 5 impressed medially	***A. alberti* Anderson, sp. n.**
–	Male with aedeagus in dorsal view tapered in apical 2/5 to narrowly rounded apex (Fig. 18); male tergite VII with apex rounded; male with apex of ventrite 5 deeply, narrowly impressed medially	***A. mensurensis* Anderson, sp. n.**
7	Male with aedeagus with endophallus extended almost to apex of aedeagal apodemes, with a narrow u-shaped sclerite at midlength of aedeagus and an apical hooked sclerite complex (Fig. 12); female with elytral profile at apical declivity distinctly angulate	***A. franklyni* Anderson, sp. n.**
–	Male with aedeagus with endophallus extended from about midlength of aedeagal apodemes to apical two-thirds of aedeagal apodemes, with either a narrow, scythe-like sclerite positioned near basal two-thirds of length, an elongate field of microtrichia positioned at base of aedeagus, and an apical hooked sclerite complex (Fig. 15), or with only an apical hooked sclerite complex (Fig. 9); female with elytral profile at apical declivity evenly rounded	**8**
8	Male with aedeagus with endophallus extended from about midlength of aedeagal apodemes to apical two-thirds of aedeagal apodemes, with either a narrow, scythe-like sclerite positioned near basal two-thirds of length, an elongate field of microtrichia positioned at base of aedeagus, and an apical hooked sclerite complex (Fig. 15); females not separable	***A. griseus* Anderson, sp. n.**
–	Male with aedeagus with endophallus extended to about midlength of aedeagal apodemes, with only an apical hooked sclerite complex (Fig. 9); females not separable	***A. beckeli* Anderson, sp. n.**

### Phylogeny and biogeographic implications

**Figure 27.**
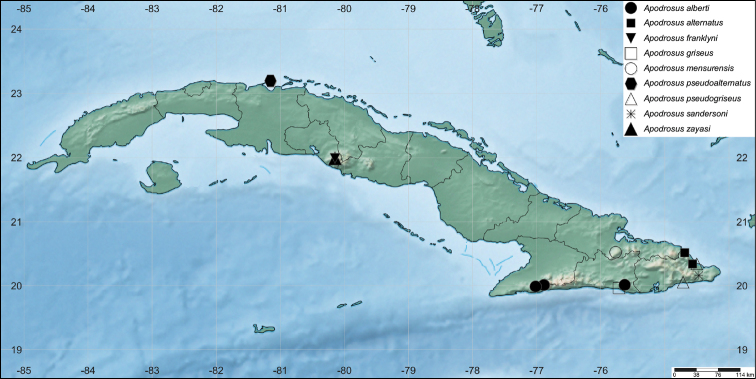
Distribution map of Cuban species of *Apodrosus*, made using simplemappr.net.

**Figure 28.**
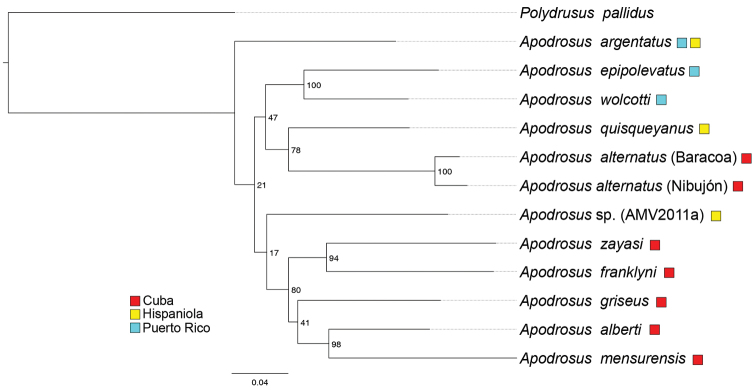
Maximum likelihood phylogeny of *Apodrosus*, based on six gene fragments. Colored boxes indicate species distributions. Bootstrap support values (1000 replications) are drawn at nodes. Hispaniola is represented by the Dominican Republic for all instances. Scale bar indicates the average number of nucleotide substitutions per site.

A distribution map of Cuban Apodrosus is shown in Fig. 27. The 33-taxon molecular phylogeny (Suppl. material 2), reconstructed using a maximum likelihood method, recovers the monophyly of *Apodrosus* (bootstrap value 100%) represented by 11 species in the current study. *Apodrosus* and *Polydrusus* are recovered as sister groups, although with poor nodal support. In other words, the monophyly of Polydrusini as sampled in the current study is recovered. It is worth pointing out that numerous genera of this tribe are not sampled here and the phylogenetic coherence or monophyly of Polydrusini remain to be fully tested. Most critically, *Anypotactus*, sister group of *Apodrosus* recovered by Girón and Franz (2010) was not sampled here. It would be premature to draw a conclusion on the placement of *Apodrosus*. *Pachyrhinus lethierryi* is nested within *Polydrusus*, necessitating the synonymy of the two genera, but this question is left for future investigations. The 13-taxon *Apodrosus* focal phylogeny is congruent with the 33-taxon phylogeny in the “deeper” relationships within *Apodrosus*, but the two differ in relationships among five Cuban species (*A. zayasi*–*A. mensurensis* clade in Fig. 28). We will base our phylogenetic and biogeographic discussions on the 13-taxon phylogeny, as our biogeographic discussions primarily concern inter-island patterns and the phylogenetic resolution within Cuban species is inconsequential in that regard.We recognize that bootstrap nodal support values are low for several clades, reflecting potential inconsistency in the data. We opted for presenting a fully dichotomous phylogeny, as is the case in many publications. Biogeographic scenarios are discussed in light of this dichotomous phylogeny (which is the best phylogeny available), but it is understood that they also are subject to future testing and revisions when additional data is acquired. *Apodrosus argentatus* Wolcott, 1924, a widespread species distributed in both Puerto Rico and the Dominican Republic, is recovered as sister to the remaining species of *Apodrosus*. The six Cuban species sampled here are not monophyletic. Five of the Cuban species form a clade (*A. zayasi*–*A. mensurensis*), which is sister to an unidentified species from Dominican Republic (which might represent a new species as it could not be keyed out using Girón and Franz 2010). A single species, *Apodrosus alternatus*, forms a sister relationship with *A. quisqueyanus* Girón & Franz, 2010, a species from the Dominican Republic. Together, they are part of a larger clade that also includes two Puerto Rican species, *A. wolcotti* and *A. epipolevatus* Girón & Franz, 2010.

The current molecular phylogeny (Fig. 28) and the morphological phylogeny by Girón and Franz (2010) differ in taxon sampling, but congruence and conflict between these phylogenies can be observed and are presented as follows. *Apodrosus wolcotti* and *A. epipolevatus*, both from Puerto Rico, are sister species in both analyses. In Girón and Franz (2010), *A. quisqueyanus* is more closely related to *A. argentatus* than either is to the two Puerto Rican species. In contrast, the current phylogeny posits a closer relationship between *A. quisqueyanus* and the Puerto Rican species, which are in the same clade, whereas *A. argentatus* is outside that clade.

According to the current phylogenetic hypothesis (Fig. 28), species of *Apodrosus* from the same island are distributed in multiple clades and some sister groups are formed by species from different islands. This pattern of the geographic distributions of species of *Apodrosus* with regards to the phylogeny is reminiscent of other Caribbean entimines (Zhang et al. 2017), and is indicative of multiple successive dispersal events between islands. A “stepping-stone” scenario of an origin in Puerto Rico and subsequent dispersal to Hispaniola and Cuba is plausible. An alternative explanation of the biogeographic pattern is island to island vicariance. According to the geological evolution model proposed by Iturralde-Vinent and MacPhee (1999), the three Greater Antillean islands, Cuba, Hispaniola and Puerto Rico, were connected to form a landspan in the Late Eocene and Early Oligocene (35–33 million-years ago [Ma]). Eastern Cuba and northern Hispaniola were physically connected during the Early Oligocene, which split apart later in that epoch. On the other hand, central Hispaniola and Puerto Rico remained connected probably until late in the Miocene (14 Ma). A sister relationship between Cuba and the Dominican Republic appears twice in our phylogeny, which is in accordance with the vicariance of the two islands. However, in the clade “*A. epipolevatus*–*A. alternatus*”, Puerto Rico is sister to Cuba and the Dominican Republic, implying an early split of Puerto Rico from the two larger islands, which is at odds with the vicariance model proposed by Iturralde-Vinent and MacPhee (1999) (i.e., a late split between Puerto Rico and Hispaniola).

## Supplementary Material

XML Treatment for Apodrosus

XML Treatment for Apodrosus alberti

XML Treatment for Apodrosus alternatus

XML Treatment for Apodrosus beckeli

XML Treatment for Apodrosus franklyni

XML Treatment for Apodrosus griseus

XML Treatment for Apodrosus mensurensis

XML Treatment for Apodrosus pseudoalternatus

XML Treatment for Apodrosus sandersoni

XML Treatment for Apodrosus zayasi

## Supplementary materials

Supplementary material 1 Information of specimens of *Apodrosus* used in molecular analyses, including Genbank accession numbers. Link: https://doi.org/10.3897/zookeys.679.12805.suppl1 Data type: Occurrence and genbank accession numbers (table). This dataset is made available under the Open Database License (http://opendatacommons.org/licenses/odbl/1.0/). The Open Database License (ODbL) is a license agreement intended to allow users to freely share, modify, and use this Dataset while maintaining this same freedom for others, provided that the original source and author(s) are credited.Robert S. Anderson, Guanyang Zhang

Supplementary material 2 33-taxon maximum likelihood phylogeny of *Apodrosus*, *Polydrusus* and representatives of other Neotropical entimines. Colored boxes indicate species distributions. Bootstrap support values (500 replications) are drawn at nodes. Hispaniola is represented by the Dominican Republic for all instances. Scale bar indicates the average number of nucleotide substitutions per site. Link: https://doi.org/10.3897/zookeys.679.12805.suppl2 Data type: Figure. This dataset is made available under the Open Database License (http://opendatacommons.org/licenses/odbl/1.0/). The Open Database License (ODbL) is a license agreement intended to allow users to freely share, modify, and use this Dataset while maintaining this same freedom for others, provided that the original source and author(s) are credited.Robert S. Anderson, Guanyang Zhang

## References

[B1] Alonso-Zarazaga MA Lyal CHC (1999) A World Catalogue of Families and Genera of Curculionoidea (Insecta: Coleoptera) (excepting Scolytidae and Platypodidae). Entomopraxis, Barcelona.

[B2] Girón JC Franz NM (2010) Revision, phylogeny, and historical biogeography of the genus *Apodrosus* Marshall, 1922 (Coleoptera: Curculionidae: Entiminae). Insect Systematics & Evolution 41: 339–414. dx.https://doi.org/10.1101/053611

[B3] Katoh K Standley DM (2013) MAFFT multiple sequence alignment software version 7: improvements in performance and usability. Molecular Biology and Evolution 30: 772–780. https://doi.org/10.1093/molbev/mst010 23329690 10.1093/molbev/mst010 PMC3603318

[B4] Marshall GAK (1922) Some injurious Neotropical weevils (Curculionidae). Bulletin of Entomolpogical Research 13: 59–71. https://doi.org/10.1017/S0007485300045247

[B5] Miller MA Pfeiffer W Schwartz T (2010) Creating the CIPRES Science Gateway for inference of large phylogenetic trees in Proceedings of the Gateway Computing Environments Workshop (GCE), 14 Nov. 2010, New Orleans, LA, 1–8.

[B6] Iturralde-Vinent MA MacPhee RDE (1999) Paleogeography of the Caribbean region: Implications for Cenozoic biogeography. Bulletin of the American Museum of Natural History 238: 3–95. hdl.handle.net/2246/1642

[B7] Stamatakis A (2014) RAxML version 8: a tool for phylogenetic analysis and post-analysis of large phylogenies. Bioinformatics 30: 1312–1313. https://doi.org/10.1093/bioinformatics/btu033 24451623 10.1093/bioinformatics/btu033 PMC3998144

[B8] Vaidya G Lohman DJ Meier R (2011) SequenceMatrix: concatenation software for the fast assembly of multi-gene datasets with character set and codon information. Cladistics 27: 171–180. https://doi.org/10.1111/j.1096-0031.2010.00329.x 10.1111/j.1096-0031.2010.00329.x 34875773

[B9] Wolcott GN (1924) “Insecta Portoricensis”. A preliminary annotated checklist of the insects of Puerto Rico, with descriptions of some new species. Journal of the Department of Agriculture of Porto Rico 7: 1–313.

[B10] Zhang G Basharat U Matzke N Franz NM (2017) Model selection in statistical historical biogeography of Neotropical weevils – the *Exophthalmus* genus complex (Insecta: Curculionidae: Entiminae). Molecular Phylogenetics and Evolution 109: 226–239. https://doi.org/10.1016/j.ympev.2016.12.039 28057552 10.1016/j.ympev.2016.12.039

