# Supplementary material for: The genus Apodrosus Marshall, 1922 in Cuba (Coleoptera, Curculionidae, Entiminae, Polydrusini)
Source: Zookeys. 2017 Jun 12;(679):77–105. doi: 10.3897/zookeys.679.12805 (PMC5523398; doi:10.3897/zookeys.679.12805)
Supplement: Supplementary material 1 — Information of specimens of Apodrosus used in molecular analyses, including Genbank accession numbers. [file zookeys-679-077-s001.docx]

Appendix 1. Locality information of specimens of *Apodrosus* used in molecular analyses and Genbank accession numbers.

| **Species** | **Catalogue number** | **Country (Region)** | **Locality** | **Latitude, Longitude** | **12s** | **28s** | **ArgK** | **COI** | **COII** | **Ef1a** |
| --- | --- | --- | --- | --- | --- | --- | --- | --- | --- | --- |
| *Apodrosus argentatus* | AM046 | Puerto Rico | Llanos | 18.01111, -67.10389 | MF101476 | MF101488 | MF101500 | HQ891422.1^*^ | MF101517 | HQ891481.1^*^ |
| *Apodrosus wolcotti* | AM047 | Puerto Rico | Jayuya | 18.17, -66.5885 | MF101477 | MF101489 | MF101501 | HQ891425.1^*^ | MF101518 | HQ891484.1^*^ |
| *Apodrosus quisqueyanus* | AM051 | Dominican Republic | Sierra de Bahoruco | 18.11261, -71.61428 | MF101478 | MF101490 | MF101502 | HQ891424.1^*^ | MF101519 | HQ891483.1^*^ |
| Apodrosus sp. (AMV2011a)^1^ | AM053 | Dominican Republic | near El Arroyazo | 19.03928, -70.51794 | MF101479 | MF101491 | MF101503 | HQ891476.1^*^ | MF101520 | HQ891535.1^*^ |
| *Apodrosus epipolevatus* | AM054 | Puerto Rico | Jayuya | 18.17, -66.5885 | MF101480 | MF101492 | MF101504 | HQ891423.1^*^ | MF101521 | HQ891482.1^*^ |
| *Apodrosus mensurensis* | ASUHIC0033601 | Cuba | Mayarí | 20.52883, -75.7683 | MF101481 | MF101493 | N/A | MF101510 | MF101522 | MF101528 |
| *Apodrosus zayasi* | ASUHIC0033636 | Cuba | Pico San Juan | 21.98868, -80.14658 | MF101482 | MF101494 | N/A | MF101511 | MF101523 | MF101529 |
| *Apodrosus franklyni* | ASUHIC0033637 | Cuba | Pico San Juan | 21.98868, -80.14658 | MF101483 | MF101495 | MF101505 | MF101512 | MF101524 | MF101530 |
| *Apodrosus alternatus* | ASUHIC0033731 | Cuba | Baracoa | 20.3506, -74.57497 | MF101484 | MF101496 | MF101506 | MF101513 | MF101525 | MF101531 |
| *Apodrosus griseus* | ASUHIC0033735 | Cuba | Siboney | 19.96227, -75.71684 | MF101485 | MF101497 | MF101507 | MF101514 | MF101526 | MF101532 |
| *Apodrosus alternatus* | ASUHIC0033736 | Cuba | Nibujón | 20.52036, -74.69018 | MF101486 | MF101498 | MF101508 | MF101515 | MF101527 | MF101533 |
| *Apodrosus alberti* | ASUHIC0034047 | Cuba | Gran Piedra | 20.001, -75.594 | MF101487 | MF101499 | MF101509 | MF101516 | N/A | MF101534 |

^1^As of May 15, 2017, the name of this species in Genbank is Polydrusini sp. 1.

^*^Downloaded from Genbank; not generated in the current study.

Gene abbreviations:

ArgK – Arginine Kinase

COI – Cytochrome c Oxidase Subunit I

COII – Cytochrome c Oxidse Subunit II

Ef1a – Elongation factor 1-alpha
