# Supplementary figures and images for: The genus Apodrosus Marshall, 1922 in Cuba (Coleoptera, Curculionidae, Entiminae, Polydrusini)
Source: Zookeys. 2017 Jun 12;(679):77–105. doi: 10.3897/zookeys.679.12805 (PMC5523398; doi:10.3897/zookeys.679.12805)

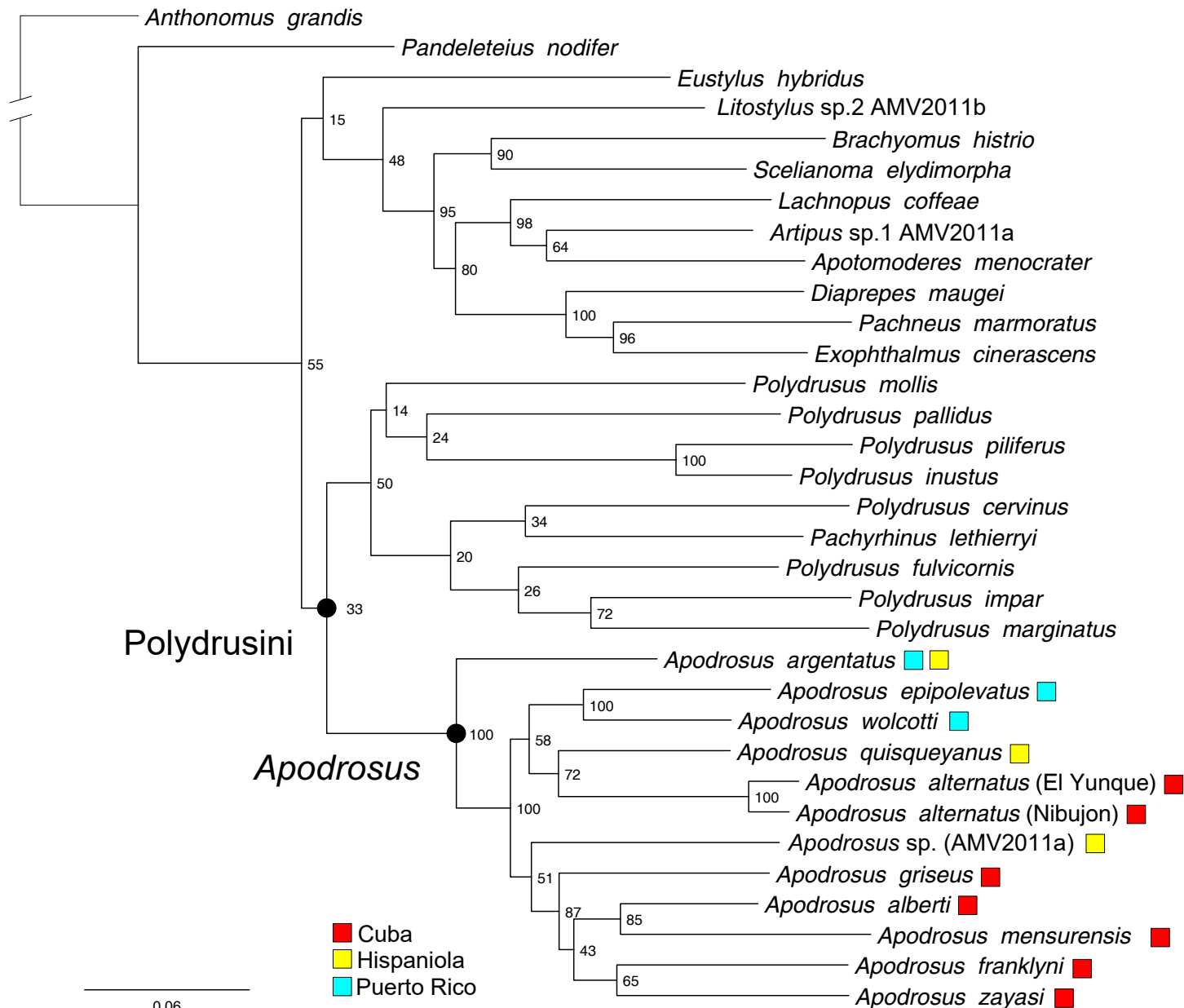

Supplement: Supplementary material 2 — 33-taxon maximum likelihood phylogeny of Apodrosus, Polydrusus and representatives of other Neotropical entimines. Colored boxes indicate species distributions. Bootstrap support values (500 replications) are drawn at nodes. Hispaniola is represented by the Dominican Republic for all instances. Scale bar indicates the average number of nucleotide substitutions per site. [file zookeys-679-077-s002.pdf]
